# Functional rehabilitation of the maxillary sinus after modified endoscopic sinus surgery for displaced dental implants

**DOI:** 10.1186/s40729-023-00490-2

**Published:** 2023-09-04

**Authors:** Buyanbileg Sodnom-Ish, Mi Young Eo, Ju Young Lee, Mi Hyun Seo, Soung Min Kim

**Affiliations:** Department of Oral and Maxillofacial Surgery, Dental Research Institute, School of Dentistry, Seoul National University, Seoul, Korea

**Keywords:** Caldwell–Luc procedure (CLP), Modified endoscopic sinus surgery (MESS), Dental implants, Endoscopic sinus surgery, Mucociliary clearance

## Abstract

**Purpose:**

Dental implants may become displaced into the maxillary sinus due to insufficient primary stability, changes in nasal air pressure, or surrounding bone resorption and should be removed as soon as possible. The aim of this study was to evaluate the efficacy of the modified endoscopic sinus surgery (MESS) approach for removal of displaced dental implants.

**Methods:**

From September 2010 to November 2021, we studied 15 cases with displaced implants in the maxillary sinus. The patient characteristics, medical history, clinical and imaging results, and post-removal outcomes were retrospectively assessed.

**Results:**

The symptoms included sinusitis (100%), pain (26.6%), postnasal drip (6.6%), nasal obstruction (26.6%), and oroantral communication (26.6%). Two cases were managed through the crestal approach (13.3%), while two cases were treated with the Caldwell–Luc procedure (13.3%). One case was addressed using functional endoscopic sinus surgery (6.7%), while 10 cases were managed with the MESS approach (66.7%). MESS allows functional rehabilitation of mucociliary clearance by the cilia in the sinus membrane. Implant displacement into the maxillary sinus can be classified as early, late, or delayed displacement.

**Conclusions:**

MESS is a reliable treatment option that can identify migrated dental implants in any part of the sinus with endoscopic assistance for functional rehabilitation of the maxillary sinus without postoperative sequelae.

## Background

Dental implants in the posterior edentulous maxilla have been widely investigated and often pose a challenge to oral surgeons due to alveolar bone resorption and maxillary sinus pneumatization [1]. The posterior maxilla is often associated with a high level of complications due to its poor bone quality and its proximity to vital anatomical structures such as the maxillary sinus. A dental implant may become displaced into the maxillary sinus for various reasons such as insufficient primary stability, changes in nasal air pressure, or bone resorption surrounding the implant. Such displacement may not be limited to the sinus floor, but may include other locations in the paranasal sinuses. The diversity of implant displacement locations warrants the use of different surgical approaches to retrieve the implants [2].

Different endoscopic and non-endoscopic approaches have been developed over the years to remove dental implants from the sinuses including the Caldwell–Luc procedure (CLP), functional endoscopic sinus surgery (FESS), crestal and upper lateral approaches under the zygomatic buttress, and a posterior lateral approach [2]. FESS uses a transnasal approach through the ostium in the middle meatus to enter the maxillary sinus and other paranasal sinuses. It allows removal of displaced implants or other foreign bodies, treatment of maxillary sinusitis, and recreation of adequate patency of the natural maxillary ostium with minimally invasive procedures. The main disadvantage of FESS is that it cannot close oroantral communications without additional intervention [1, 3].

In contrast, an intraoral approach allows for removal of displaced dental implants and closure of oroantral fistulas with local flaps such as buccal, palatal, and rotational palatal flaps. However, this method is not an effective treatment for an obstructed osteomeatal unit (OMU) or eventual sinusitis of the paranasal sinuses.

In the literature, no single ideal approach was established for the removal of displaced dental implants in the maxillary sinus. Recently, a new method called modified endoscopic sinus surgery (MESS) was introduced to treat odontogenic maxillary sinusitis, maxillary retention cysts, blow-out orbital fractures, ectopic third molars in the maxillary sinus, apicoectomies of dental implants, and removal of implants beneath the optic canal [4–6]. The aim of this study was to review the timing and its associated symptoms of dental implant displacement into the maxillary sinus and to evaluate the efficacy of the MESS approach in managing implant displacement.

## Methods

We performed an 11-year retrospective review of patients who presented to the Department of Oral and Maxillofacial Surgery at Seoul National University Dental Hospital from September 2010 to November 2021 and studied 15 cases with displaced implants in the maxillary sinus. The study protocol complied with the principles of the Declaration of Helsinki and was approved by the Seoul National University Institutional Review Board (S-D20170005). All methods were performed in accordance with the relevant guidelines and regulations. All patients were informed of the surgical procedure with the potential risks and benefits, and an informed consent was obtained to undergo treatment and be included in the study.

**Fig. 1:**
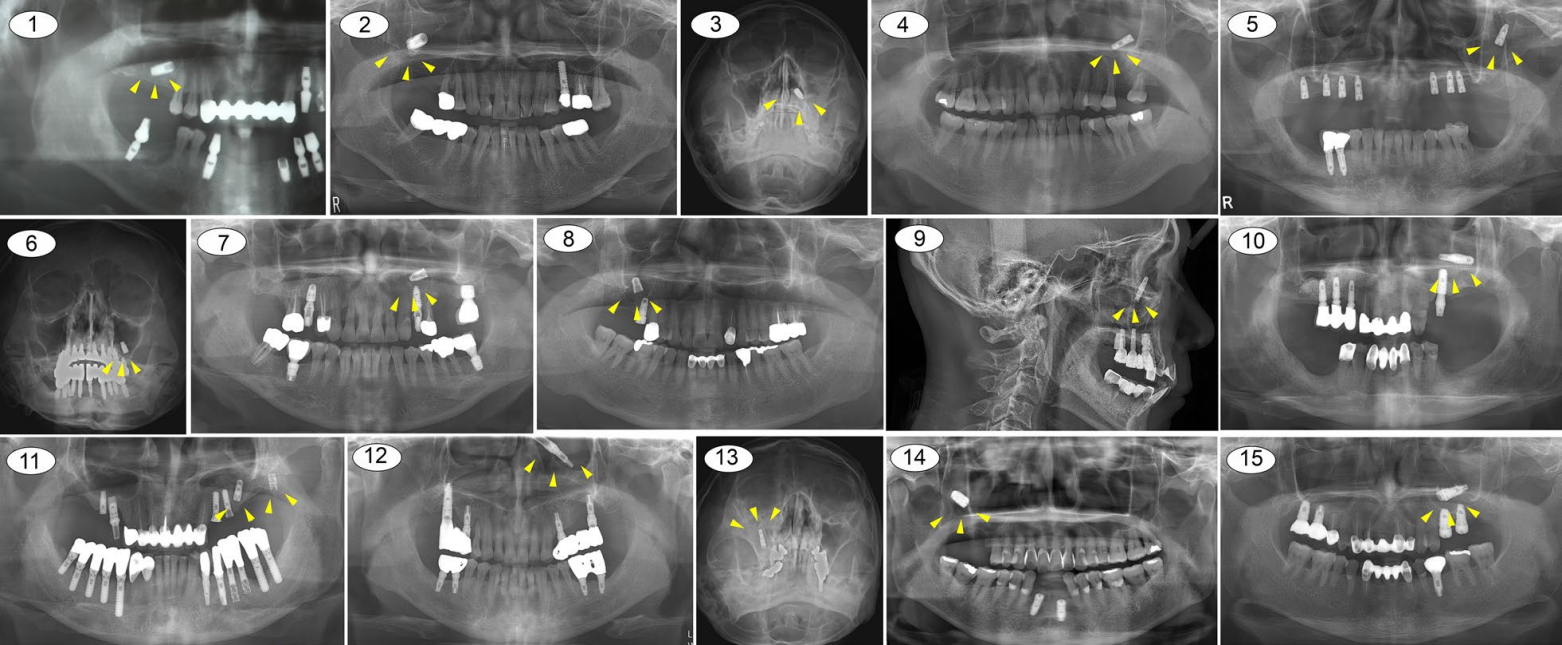
The preoperative radiographic evaluation visualizing displaced dental implants on panoramic radiographs, Water’s view, posteroanterior and lateral cephalograms, and computed tomography of the 15 patients included in this study

Implant migration was identified in 8 male and 7 female patients with an age range of 48–74 years. The patient characteristics, medical histories, clinical and imaging results, and post-removal outcomes were retrospectively assessed. Two patients presented within the first 24 h of displacement, 10 patients presented within the first 8 weeks of implant placement, and 3 patients presented 6 months after loading. All patients had unremarkable medical histories. The patient demographic information is shown in Table 1. Panoramic radiographs, Water’s view, and paranasal sinus (PNS) computed tomography (CT) were used to investigate the anatomical locations of the displaced dental implants. There were 6 cases of implant displacement in the right maxillary sinus (40%) and 9 cases in the left maxillary sinus (60%) (Fig. 1).

**Table 1:** Demographic characteristics of the patients included in the study

Patient no.	Gender	Age	Symptoms	Past medical history	ASA	Location	OMU obstruction	Oroantral communication
1	M	70	Sinusitis, pain	Not specific	I	Rt. Mx. Sinus (#17i)	Not present	–
2	F	59	Sinusitis	Not specific	I	Rt. Mx. Sinus (#16i)	Present	Present
3	F	62	Sinusitis	Not specific	I	Lt. Mx. Sinus and ethmoid sinus (#27i)	Present	Present
4	M	51	Sinusitis, pain	HTN, DM, HBV	II	Lt. Mx. Sinus (#26i)	Present	Present
5	M	64	Sinusitis	Not specific	I	Lt. Mx. Sinus (#26i)	Not present	Present
6	M	57	Sinusitis	Not specific	I	Lt. Mx. Sinus (#26i)	Not present	Present
7	F	48	Sinusitis, nasal obstruction	HTN	II	Lt. Mx. Sinus (#26i)	Present	Present
8	F	70	Sinusitis, pain	HTN	II	Rt. Mx. Sinus (#17i)	Present	–
9	M	66	Sinusitis	Not specific	I	Rt. Mx. Sinus (#16i)	Not present	–
10	M	57	Sinusitis, nasal obstruction	DM	II	Lt. Mx. Sinus (#26i)	Present	Present
11	M	74	Sinusitis	HTN, Heart disease	II	Lt. Mx. Sinus (#26i, 27i)	Not present	Not present
12	M	64	Nasal discharge, nasal obstruction	DM, Bleeding tendency	II	Lt. Mx. Sinus and nasal cavity (#26i)	Present	Not present
13	F	71	Sinusitis	Osteoporosis	II	Rt. Mx. Sinus (#17i)	Present	Present
14	F	55	Sinusitis	Not specific	I	Rt. Mx. Sinus (#16i)	Not present	Present
15	F	60	Sinusitis, pain, nasal obstruction	Not specific	I	Lt. Mx. Sinus (#27i)	Present	Present

*L/C* local clinic, *HTN* hypertension, *DM* diabetes mellitus, *HBV* hepatitis B virus, *ASA* American Society of Anesthesiologists score, *Rt* right, *Lt* left, *Mx* maxilla, *OMU* osteomeatal unit

Four main surgical approaches were used to retrieve the displaced dental implants: (1) crestal approach through the implant insertion site; (2) a CLP through the bony window near the canine fossa; (3) FESS through the nasal cavity for identification and enlarging of the OMU, which is the opening part of the maxillary sinus and ethmoidal sinus; and (4) MESS. All surgeries were carried out by one experienced surgeon. The MESS technique, which involves accessing the maxillary sinus through the lateral window between the canine fossa and the buttress, was developed and refined over time based on the CLP and FESS approaches.

### Classification

The patients were retrospectively divided into three groups based on the timing of implant displacement: early displacement, late displacement, or delayed displacement (Table 2). The early displacement group included all complications that occurred intraoperatively and were associated with incorrect surgical planning, including placement of implants at sites with inadequate bone height and volume, a lack of surgical experience, overpreparation of the recipient site, applying heavy force during implant insertion, and sinus membrane perforation during the drilling procedure. Late displacement was related to implant displacements that occurred during the follow-up and re-entry procedures and typically occurred within the first 6 months resulting from incorrect surgical technique, constant bone destruction due to an existing alveolar bone infection, osteoporosis, or osteopenia [7, 8]. Delayed displacement was observed in all cases after prosthesis delivery due to changes in paranasal pressure, destruction of the bone around the implant leading to impaired osseointegration, resorption resulting in the incorrect distribution of occlusal forces, and detachment of the implant from the prosthetic retention structure [9].

**Table 2:** Classification of implant displacement based on timing

Timing of displacement	Criteria of displacement	No. of patients	%
Early displacement	Implant displacement occurring intraoperatively before osseointegration	2	13.33
Late displacement	Implant displacement occurring during follow-up and re-entry, usually within six months	3	20
Delayed displacement	Implant displacement occurring after prosthesis delivery	10	66.67
Total		15	100

### Surgical technique

Antimicrobial prophylaxis was administered using 250 mg cephalosporin, 386 mg ibuprofen arginine, and 95 mg Phazyme taken three times a day for 5 days. All of the procedures were carried out under general or conscious sedation using midazolam. A 2% lidocaine solution with 1:100.000 epinephrine was infiltrated into the buccal sulcus of the affected site. Epinephrine and xylocaine-soaked cotton were applied in an alternating manner to the middle meatus for 10 min before local anesthetic administration. The MESS technique can be performed under local anesthesia for the removal of a displaced dental implant in the maxillary sinus. However, in cases of extensive maxillary sinusitis or more complex procedures, intravenous sedation may be required.

**Fig. 2:**
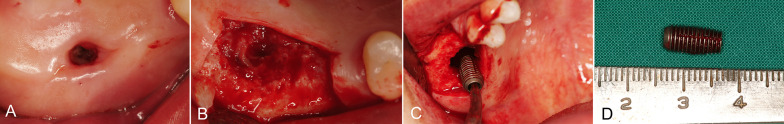
The crestal approach for displaced implant removal through the implant insertion site in Case 2. Preoperative intraoral view (**A**), a full-thickness mucoperiosteal flap (**B**), displaced implant removal using the suction tip (**C**), and the removed implant specimen (**D**)

The crestal approach was established by a horizontal incision following the margins of the oroantral communication (if present) and extended distally and mesially with releasing vertical incisions. A full-thickness mucoperiosteal flap was elevated, and the implant was removed from the maxillary sinus using a thin suctioning tip and/or sinus forceps through the implant insertion site (Fig. 2A–D).

**Fig. 3:**
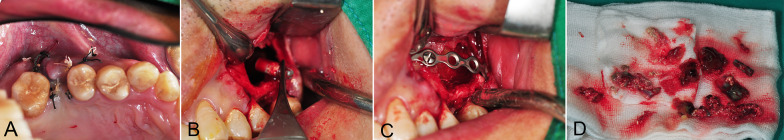
The CLP approach to access the displaced implant and infected bone graft materials in Case 4. Preoperative intraoral view (**A**). After implant removal, the maxillary sinus was carefully irrigated (**B**). In cases of extensive destruction in the maxillary anterior wall, a 6-hole miniplate was used for reconstruction (**C**). Inflamed tissue, infected bone graft material, and the displaced dental implant specimen (**D**)

For the CLP procedure, a crestal incision followed by a full-thickness mucoperiosteal flap was raised with the aim of exposing the anterior-lateral wall extending from the canine to the molar region. A 1.5-cm window was ground into the anterior wall. The Schneiderian membrane (SM) was incised for removal of the displaced dental implant, and the pathologic mucosa was enucleated with a surgical curette. After implant removal, the maxillary sinuses were carefully irrigated and the SM (if present) was sutured using 6–0 Vicryl® (Polyglactin 910; Johnson & Johnson, Somerville, NJ, US). In cases where there was extensive destruction of the maxillary anterior wall, a 6-hole miniplate was used for reconstruction (Fig. 3A–D).

**Fig. 4:**
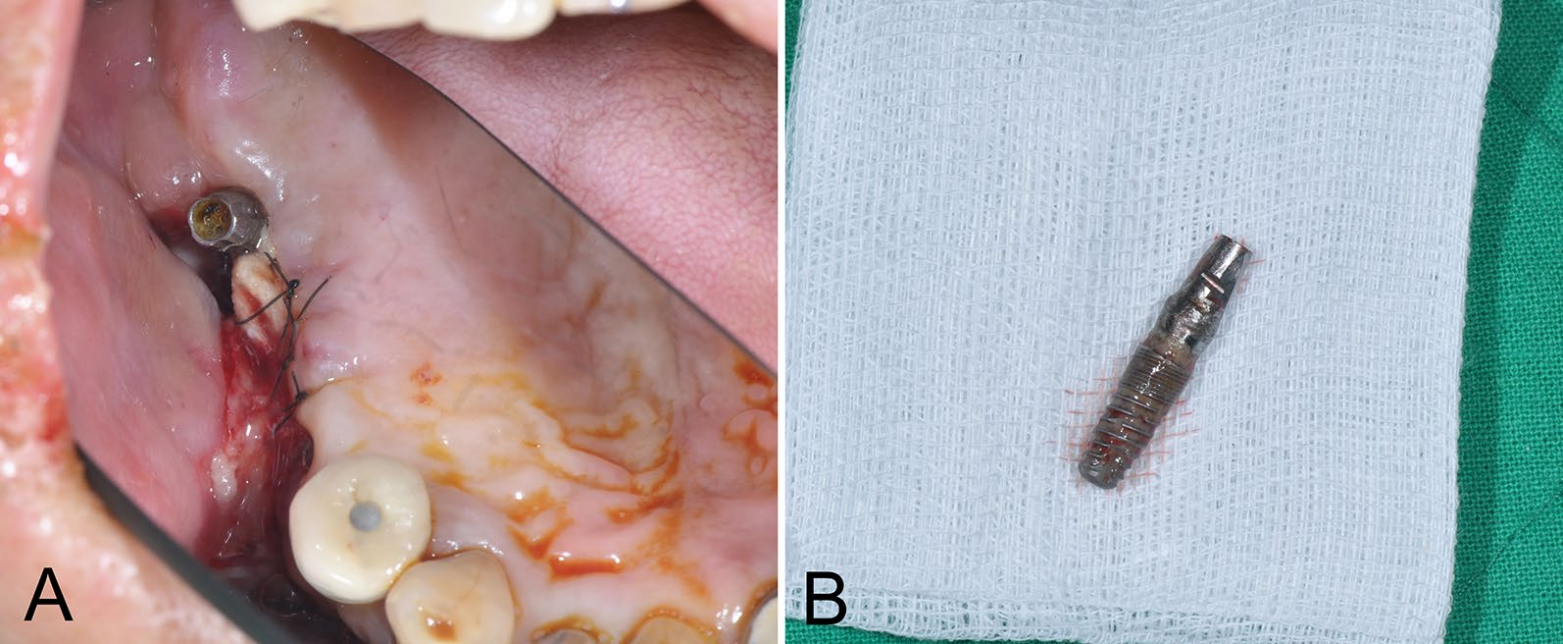
Preoperative intraoral view of Case 9 for FESS (**A**). The removed #16i displaced dental implant specimen (**B**)

The FESS was used in cases of implant displacement in the maxillary sinus with or without paranasal sinusitis symptoms and OMU obstruction, but with no oroantral communications. The endoscopic technique included a partial uncinectomy and enlargement of the maxillary sinus ostium in a middle meatal antrostomy, allowing for easier access to the maxillary sinus and reestablishment of adequate patency to the OMU. Hemostasis was established with diathermy intraoperatively and an anterior nasal tamponade at the end of the surgery (Fig. 4A, B) [3].

**Fig. 5:**
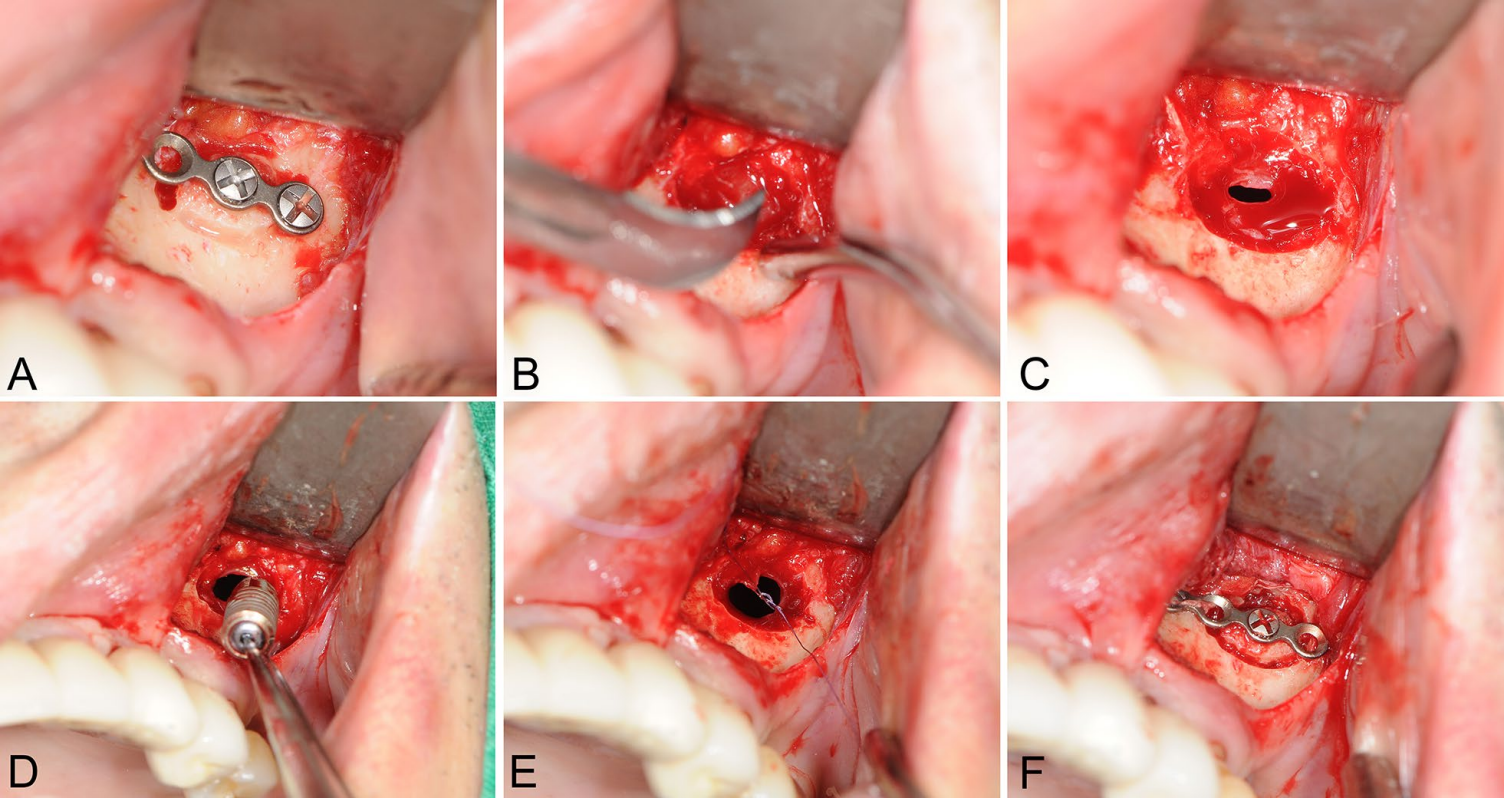
The routine MESS procedure consisted of creating a pre-fixed 10 × 7 mm bony window on the anterolateral wall of the maxillary sinus using a small round bur with a diameter of 0.5 mm, as shown in Case 5 (**A**). The Schneiderian membrane was incised minimally using a sharp scalpel, followed by endoscopic assessment of the inner surface of the maxillary sinus (**B**). To approach the implant, a firm and gentle negative suction force was applied through the sinus bony window, while visualization and illumination were obtained from the nasal meatal endoscope (**C**). After implant removal, the sinus was carefully irrigated with warm saline, and the Schneiderian membrane was re-sutured (**D**, **E**). The bony window was repositioned into its original position and was fixed with a four-hole miniplate and pre-drilled screws (**F**)

**Fig. 6:**
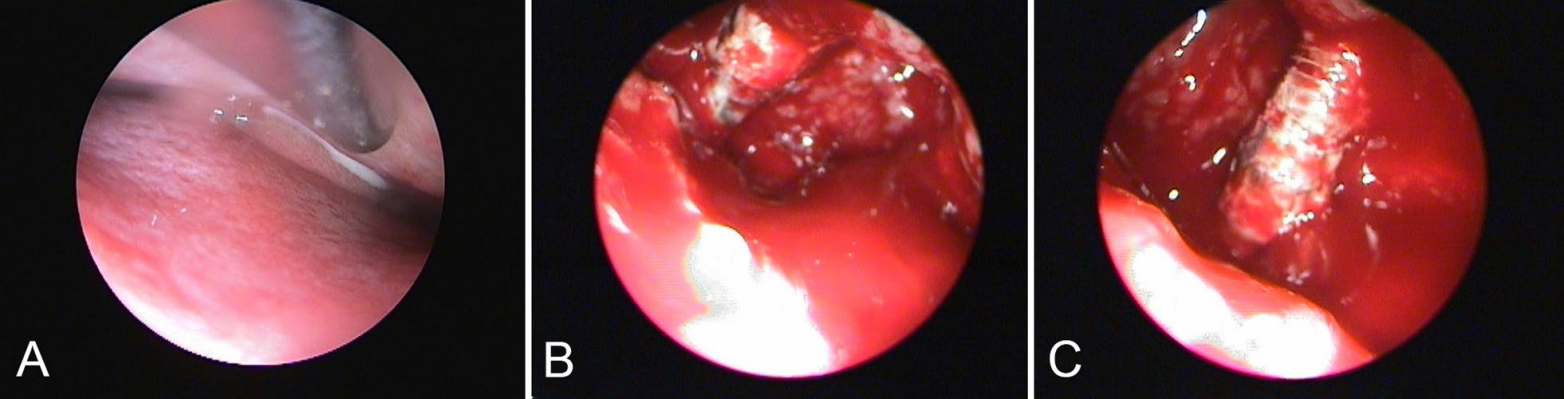
Intraoperative endoscopic images of the displaced dental implant. Endoscopic inspection though the nose and osteomeatal unit (**A**). Endoscopic inspection though the upper maxilla after creating a bony window (**B**, **C**)

The MESS approach included a vestibular incision followed by the creation of a bony window 10 × 7 mm in size in the anterolateral wall of the maxillary sinus. The bony window was stabilized with a four-hole microplate and partially fixed to minimize the time required for repositioning. The bony window was then separated, and the SM was carefully incised with a scalpel (Fig. 5A–C). Several different (0º, 70º, and 90º) endoscopes were used to inspect the maxillary sinus though the incision (Fig. 6A–C). The inflamed tissue and displaced dental implants were then removed using a thin suctioning tip and/or with sinus forceps. After sinus irrigation, the SM was sutured using 6-0 Vicryl® (Polyglactin 910; Johnson & Johnson, Somerville, NJ, US.). The bony window was repositioned and stabilized using a 4-hole microplate and screws (Fig. 5A–F).

## Results

The signs and symptoms presented by patients with implant displacement included sinusitis (100%), pain (26.6%), postnasal drip (6.6%), nasal obstructions (26.6%), and oroantral fistulas (26.6%). Two patients were managed through the crestal approach (13.3%), while two cases were addressed using the CLP technique (13.3%). One patient was treated with the FESS approach (6.7%), and the remaining 10 cases were managed with the MESS approach (66.7%) (Table 3).

**Table 3:** Summary of the surgical approaches used in this study

Patient no.	Anesthesia	Surgical procedure	Biopsy results	Follow-up period after treatment
1	IVS	Crestal approach	–	3-month follow-up
2	IVS	Crestal approach	–	3-month follow-up
3	GA	CLP	Chronic maxillary sinusitis	2-year follow-up
4	IVS	CLP	1. Chronic maxillary sinusitis 2. Foreign body reaction around the graft material	3-year follow-up
5	IVS	MESS (lateral bony window)	–	3-year follow-up
6	IVS	MESS (lateral bony window)	–	1-year follow-up
7	GA	MESS (lateral bony window)	Chronic maxillary sinusitis	2-year follow-up
8	IVS	MESS (lateral bony window)	–	6-month follow-up
9	IVS	FESS	–	3-month follow-up
10	GA	MESS (lateral bony window)	Chronic maxillary sinusitis	4-year follow-up
11	IVS	MESS (lateral bony window)	Chronic maxillary sinusitis with a fungus ball (aspergillosis)	4-year follow-up
12	IVS	MESS (lateral bony window)	Chronic maxillary sinusitis	1-month follow-up
13	IVS	MESS (lateral bony window)	Chronic maxillary sinusitis	2-month follow-up
14	IVS	MESS (crestal opening)	Chronic maxillary sinusitis	2-year follow-up
15	IVS	MESS (crestal opening)	1. Inflamed fibrous tissue 2. Inflamed granulation tissue 3. Foreign body materials	2-year follow-up

*IVS* intravenous sedation, *GA* general anesthesia, *CLP* Caldwell–Luc operation, *FESS* functional endoscopic sinus surgery, *MESS* modified endoscopy-assisted sinus surgery

**Fig. 7:**
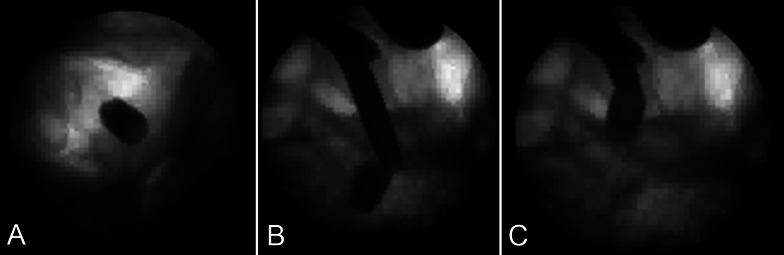
The dental X-ray fluoroscopic equipment DreamRay 60F® (DreamRay, Pusan, Rep. of Korea) was used to identify the location of the displaced implant in a severely superior and posterior location of the sinus. Grasping and suction were used with the “naked eye” to approach and remove the implants in Case 1 (**A**–**C**)

Regarding the timing of displacement, two cases occurred in the early displacement period (13.33%), three cases were in the late displacement period (20%), and 10 cases arose in the delayed displacement period (66.67%). Based on our experience with Cases 1 and 2 where dental X-ray fluoroscopic equipment DreamRay 60F® (DreamRay, Pusan, Korea) was used to identify the exact location of the displaced implant in a severely superior and posterior location of the sinus, grasping and suction were applied using the “naked eye” to approach and remove the implants (Fig. 7A–C). This method resulted in a relatively large oroantral communication. Therefore, to prevent this outcome, we developed the simple and efficient MESS approach where the exact location of the implant in the sinus can be identified using endoscopic assistance [6]. In Case 3 where the implant was displaced into the left maxillary and ethmoid sinuses, an FESS approach was initially attempted, but the implant could not be identified or accessed. Therefore, a CLP approach was planned.

Nine of the 15 cases exhibited OMU obstruction. In addition, 10 cases had oroantral communication while two did not (Table 1). The hospitalization period ranged from one to three days for the patients who underwent conscious sedation and general anesthesia. The patients were followed at 1 week, 1 month, 3 months, 6 months, and 12 months after the surgery. The success rate of the MESS procedure was 100% (10 cases), and patients exhibited an uneventful complete recovery of the paranasal sinuses, disappearance of the signs and symptoms of sinusitis, and functional maxillary sinus rehabilitation without any recurrence in the clinical and radiographic findings.

Patient satisfaction has not been verified under any regulated criteria, but most of the patients were satisfied with their surgical treatment and recovered promptly with a short hospital stay under conscious sedation without general anesthesia. In addition, they exhibited preservation of nasal cavity physiologic airflow and maintained maxillary sinus function while avoiding volume loss by retaining lateral window repositioning.

## Discussion

Dental implant therapy has become a widespread procedure performed by many dentists and oral surgeons. As the frequency of such treatment increases, the number of complications associated with implant placement will also increase [10]. Dental implant displacement into the maxillary sinus is a complication that can result in maxillary sinusitis, OMU obstruction, nasal obstruction, foreign body aspiration, bony necrosis, and migration into deeper cavities including the ethmoid and sphenoid sinuses, the orbit, the anterior cranial fossa, and the stomach [10]. For these reasons, displaced dental implants should be carefully removed as soon as possible to prevent any further damage [2]. In the current study, different surgical approaches for the removal of displaced dental implants and maxillary sinusitis treatment were discussed along with their limitations and advantages.

CLP has been considered the most favorable approach to the maxillary sinus for its ease of access and visibility. However, several complications, such as postoperative maxillary cysts and a high recurrence rate of sinusitis symptoms, which are associated with inferior osteotomy have been reported [3]. In addition, CLPs alone cannot resolve OMU obstructions due to maxillary sinusitis. By comparison, the crestal approach has more direct access to the sinus and is less invasive. Although it causes less surgical trauma, this method is a blind procedure and may require the socket to be enlarged to remove the displaced dental implant trapped in the undercut of the sinus, which may lead to postoperative depression of the alveolar ridge [2].

The FESS approach has been considered the best modern route due to its decreased invasiveness compared to the CLP approach. To access the maxillary sinus, three approaches exist: the standard transnasal endoscopic approach, the inferior meatal antrostomy, and the transnasal endoscopic approach [10–12]. Although these transnasal endoscopic approaches may appear to allow easy access to identify and remove the foreign body from the maxillary sinus, it can be difficult to remove or even identify displaced dental implants that are located in the anteroinferior part of the sinus [7, 11, 13, 14]. In contrast, the MESS approach can overcome this complication and identify the migrated dental implant in any part of the sinus with endoscopic assistance through a bony window placed in the anterolateral wall [5, 6].

In the recent literature, a transoral endoscope-assisted approach has been introduced for removal of displaced dental implants in the maxillary sinus due to its wider visibility and access compared to the transnasal approach [11]. The authors suggested that a transoral endoscope-assisted approach could be successful in all cases except those with ostium obstruction or a structural abnormality of the OMU. One of the main advantages of the MESS approach is that it allows an assessment of the patency of the ostium and anatomical abnormalities of the surrounding structure with the use of an intranasal endoscope examination along with enlargement of the ostium using sinus forceps or curved Kelly forceps [5, 6, 15]. MESS is a powerful alternative to conventional CLP and FESS procedures as it preserves the natural functions of the paranasal sinus while maintaining the sinus anatomy. MESS is performed by creating a lateral window on the posterior aspect between the canine fossa and buttress, where a form is created according to the originating tooth, lateral cortical bony thickness, and alveolar antral artery location.

MESS offers various means to avoid postoperative sinus scar tissue or the formation of POMC. One effective method is repositioning the bony window to its original position. Unlike traditional CLPs where the SM may adhere to the oral mucosa, the MESS procedure avoids this issue by utilizing autogenous bone with osteoinductive and non-immunogenic properties. This eliminates the need for additional membranes to isolate the maxillary sinus and prevents soft tissue migration into the sinus cavity. The use of a microplate to secure the bony window further enhances stability and bone healing.

Regarding the removal of a displaced dental implant from the maxillary sinus, FESS alone may not be adequate as it mainly focuses on treating sinus-related issues and does not specifically address the removal of foreign objects from the sinus. In contrast, the MESS technique is specifically designed to address situations like removal of a displaced dental implant from the maxillary sinus, making it the more suitable choice for this specific odontogenic issue.

Compared to performing FESS and CLP under general anesthesia, MESS is less invasive in terms of anesthesia requirements. Local anesthesia or intravenous sedation in MESS can provide sufficient comfort for the patient without the need for general anesthesia, making it a more favorable option for certain cases. This reduced invasiveness can lead to shorter recovery times and potentially fewer complications. However, the choice of anesthesia should always be based on the patient's individual medical history, the extent of the procedure, and the surgeon's assessment.

Based on our findings, the MESS approach could be indicated in the following cases of displaced dental implants in the maxillary sinus:

1. Cases with no previous lateral window opening.
2. Displaced implants in difficult anatomical locations where the FESS and the crestal approaches would not be beneficial.
3. Cases aiming for functional rehabilitation of the mucociliary clearance by the cilia in the sinus membrane following MESS.

## Conclusions

Based on a literature review and the current clinical findings, implant displacement into the maxillary sinus could be classified as early, late, or delayed. The MESS procedure is a reliable treatment option for functional rehabilitation of the maxillary sinus without postoperative sequelae in cases of implant displacement regardless of its location in the sinus.

## Data Availability

The datasets used and/or analyzed during the current study are available from the corresponding author on reasonable request.
